# Statement of Fifteen Cases of Midwifery in Which Chloroform Was Administered

**Published:** 1848-11

**Authors:** Geo. N. Burwell

**Affiliations:** Buffalo


// BUFFALO MEDICAL JOURNAL

AND

MONTHLY REVIEW.

VOL. 4. NOVEMBER, 1848. NO. 6.

### ORIGINAL COMMUNICATIONS.

ART. 1.—*Statement of fifteen Cases of Midwifery in which Chloroform was Administered.* By Geo. N. Burwell, M. D.

I have administered chloroform in fifteen cases of midwifery. The time I have kept my patient, more or less continuously, under its influence has varied, in the different cases, from five minutes to one and a half hours. I shall give the cases somewhat in detail, even at the risk of being tedious; certain that by so doing, I can give a much more just idea of them, not only for comparison with other cases, but as a guide to those who may also feel inclined to administer this medicine.

The cases are susceptible of division into two classes, according to the degree of anæsthesia produced: 1st, those in which it was total or nearly so: 2d, those in which a perfect knowledge of everything going on was retained; or at least, only momentarily lost.

Eight of the cases may be included under the first division, as follows:

1. Mrs. L.—I was called at 11 1-2 P. M., Feb'y. 19, last. I heard her screams from the street, before I had entered the house. The waters had discharged at 8 o'clock, since which time the pains had been violent, and bearing down with intervals of only about three minutes. Her pulse was 100, skin warm and moist. The labia were largely swollen, dry and hot; the head had reached the inferior strait, and the vertex covered with an œdematous tumor. During the pains it was forced down so closely against the ischia, that the finger could not be passed beyond these bones; in the intervals of pain it receded so that the position could be readily made out. I, at first, expected to effect a rapid delivery by the free use of fresh lard, and making moderate pressure upon the perineum during the pains. Finding no progress made after five or six pains, and the woman's screams so violent, I had her inhale chloroform from a small bit of sponge. Twenty or thirty drops only were poured upon it, and, covered with a piece of oiled silk, it was held to her nostrils. This amount of chloroform answered for two and sometimes three pains; no insensibility was at this time produced by it, but it had the effect of easing the pains so that she did not make so great an outcry; yet there was the same violent effort, the same rapid and forcible descent of the head against the ischia, and the same retrocession during the intervals. Forty minutes having elapsed since my arrival, and no progress made, and feeling that I could safely deliver her in a few minutes with the forceps, I sent for them, and continued the use of the chloroform to a point short of insensibility, until they were brought to me. Thirty minutes more had now been spent; and it was during this time that on the accession of pains she repeatedly called for "some more of that stuff which made such a fool of her," and which, often, was the first intimation I had of pain being about to come on.

Having her placed across the bed in a position convenient for the application of the forceps, I saturated the sponge with the chloroform, (which took very nearly a fluid drachm,) and had her breathe freely of it, and in about a minute she was fully under its effects, lying perfectly quiet, relaxed and unconscious. I had its breathing continued a few respirations after this, while I applied the forceps, and quickly and readily delivered the head. By slight traction on this, the shoulders and body soon followed, and the womb contracted down well. She lay, in the mean time, in a full sleep, and perfectly unconscious of what was going on. There was moderate involuntary bearing down when I made traction on the head. The cord was tied and separated. On examination, the after-birth was found to be wholly within the womb, so no effort was made to extract it. Some four or five minutes afterwards, she still lying in a quiet slumber, slight traction was made on the cord by grasping it with the left hand, while the right fore-finger was carried to, or near its junction with the placenta; and the latter was found to yield, and soon came away with a number of clots of blood. Just at this moment she suddenly came to herself, and cried out, so we knew that she was awake again.

There was considerable flooding, accompanied with sharp after-pains, for which the usual remedies were sufficient. The next morning her pulse had fallen to 80. On the third and fourth days, as the milk came, it rose to 116, and was accompanied with considerable pain and soreness in the lower part of the bowels. This condition was relieved by a dose of oil, followed by Pulv. Doveri, gr. vi., q. h. 4, warm fomentations, teas, &c. This was her third child.

2. Mrs. J.—Was called at 2 1-4 A. M. Found her having the ordinary dilating pains, with the fourth child. She is of full habit, and has a general expression of uneasiness. At 5 o'clock there was an interval of ten minutes between each pain. The os uteri dilated to ½ inches in diameter, and grew thin, and fitted closely to the head during a pain; small bag of waters; presentation 1st vertex, and the head fairly in the cavity of the pelvis. At 6 o'clock the pains continued of good strength, causing considerable outcry during their continuance. But little progress of dilatation—V. S. to 20℥.

At 7 o'clock commenced the inhalation of chloroform; at first allowing but a few moderate inhalations just at the commencement of a pain, and occasionally letting a pain go over without administering it. The pains were good and the dilatation making good progress. At 20 minutes past 7, ruptured the membranes. The inhalations were now allowed to be freer. I was inclined to think that the pains were not so hard or expulsive. She certainly did not feel them much, although not yet insensible—said that the inhalations "*benumbed*" the pains. At 7 1-2, finding the head beginning to distend the perineum, I directed still fuller inhalations; that is, the sponge was kept more moist with chloroform than at first, and it was breathed a larger number of times during each pain; the head came down naturally; there was no outcry; and finally, at 20 minutes to 8, the child was born; none of the assistants or bystanders thinking her at all sensible or knowing to its birth. She then lay for five minutes or more as if in a quiet slumber. At the delivery of the after-birth she made considerable complaint, I being obliged to deliver it manually, it being caught behind the pubes. Her after-pains were rather violent, and she had some sickness at the stomach within an hour, caused by the chloroform very likely; there were no more than the ordinary lochia; no milk fever or other bad symptom to retard a perfect recovery.

She had some dizziness about the head during the day when rising upon her elbow—none if lying quiet; no headache. She thinks she had some faint idea of the birth of the child at the moment of its passage into the world. On waking from the effects of the chloroform she felt a sense of oppression, as though the room was too confined, but not to the extent of complaining about it, or speaking of it until especially asked about her feelings.

3. Mrs. J.—On answering the summons to see her, I found her in labor with her second child; os uteri dilated; the waters escaped; and the head well advanced in the cavity of the pelvis. The pains were rapid, regular, and forcible, causing a good deal of suffering. She at once consented to breathe the chloroform, which I gave in full doses; that is, two-thirds of a teaspoonfull was poured on to the sponge; this lasted for three or four pains, when the sponge was again wet. I gave it until I noticed relaxation in her arms, as she grasped the sheet tied to the bed post; her limbs were simultaneously relaxed; the abdominal muscles continued to act, but with diminished power. Her mind was wandering; she kept calling her husband's name, thus shewing a strong contrast in this particular with the two first cases, which did not exhibit the least mental aberration, but lay as if asleep. In twenty minutes from the commencement of the administration of the chloroform, she was safely delivered without pain or suffering. Not a single untoward symptom retarded her recovery. The after-pains were very slight.

4. Mrs. D.—I was called at 9 o'clock, and found her in labor with her first child. She is twenty four years of age. Has been in pain since 5 o'clock. They are rapid, and accompanied with a good deal of apparent distress; face flushed, pulse 84 and and active, skin warm. Has been very well and hearty to eat. V. S. to 18℥; blood buffed and moderately cupped. The pains continued sharp, but short, after the bleeding, and at the time they were on, caused strong outcries. The dilatation of the os uteri proceeded well, at 1-4 to 11 it having attained its full dilatation, and the head had become well advanced into the cavity of the pelvis. There was a disposition to doze between the pains; she would not bear down well, and was fearful and impatient. Hoping to relieve this condition so that she would exert herself more, I gave chloroform in moderate doses, principally during the intervals of pain; the pains coming on so sudden, and were so short, that I could not follow my usual plan of giving it on their accession. The pains were at intervals of five minutes. The result was not satisfactory, except in relieving her outcries. She said that she never once became insensible during the three-fourths of an hour I continued the inhalation at short intervals, nor did she think that it relieved her much.

The head had advanced, in the meanwhile, nearly to the perineum. During the next one and a quarter hours, the character of the pains did not change; she dozed when free from them, and was entirely sensible; pulse 84, regular.

At 20 minutes to 1, the head began to protrude through the vulva, causing a decided increase of suffering. The pains followed each other at intervals of only two or three minutes, and were much more expulsive in character. In ten minutes more the head engaged in the vulva without withdrawing itself during the intervals of pain, and I directed inhalation of the chloroform in a full dose. The vulva soon became greatly distended, and its edges very thin, requiring constant support to prevent the danger of laceration; thus pain followed pain for fifteen minutes, before the head finally escaped into the world. She cried out with each pain, and more during the last pain than with the others, but not to the degree she did for the ten minutes before I commenced the inhalation the last time. I supposed her, however, to be entirely sensible, and was somewhat surprised on her telling me the next day, that she did not recollect anything concerning the labor or birth of the child after commencing to breathe the chloroform the last time. She certainly, during the delivery of the head and shoulders, made quite an outcry, and bore down precisely as a woman does, not under the influence of chloroform. The child was nearly asphyxiated from having the cord once tightly around its neck, and it being kept thus tightened during the engagement of the head in the vulva. She had a good getting up for a fortnight, but then got a sore breast, which caused her a good deal of trouble. There were neither after-pains nor flooding; nor any headache nor dizziness nor other symptom, that could by possibility be referred to the chloroform.

5. Mrs. D.—1st child. Was called at 6 o'clock, P. M. She had been under the care of another physician since morning. I understood from him that the head had reached the inferior strait about noon, but since then had not advanced any. She was quite hoarse from her cries; the pains were feeble, and returned at intervals of six or eight minutes. The head occupied the inferior strait, and presented an unusually large soft flaccid tumor, evidently from the effusion of blood under the scalp. There was no use of delay, and as the forceps afforded a better chance of a speedy and safe delivery than ergot, it was determined to use them. She was properly placed in bed, about a fluid drachm of chloroform poured on the sponge, and she directed to breathe of it.

The inhalation and delivery were precisely similar to that detailed in the first case, and need not be here repeated. There were no muscular efforts, no crying out; the pulse and respiration regular and equal, and the patient appearing as if in a quiet slumber. The child was still born, a result generally to be predicted, I believe, when we meet with such a cephalic tumor, so large, and so entirely without tenseness. Her recovery was rapid and complete.

6. Mrs. K.—She had some severe pain a fortnight before her confinement, which, I felt, threatened premature delivery. The morning of the day on which she was confined, she sent for me on account of an attack of diarhoea and pain. By noon both were arrested, and she lay entirely comfortable until 5, P. M., when some pain in the back came on, preceded by some flowing; the pains increased, and soon became regular labor pains. I was sent for at 8 1-2 o'clock in the evening. The pains returned regularly every five minutes; os uteri dilated to an inch in diameter. I left her to return at 1 4 before ten. Os uteri then nearly two inches in diameter; bag of waters well formed; presentation of child 1st position of the vertex. From 10 to 10 1-4 o'clock I counted the pains; there were six of them, lasting each from one to two minutes. I then commenced the administration of chloroform in moderate doses. She breathed it with the greatest avidity; held the sponge herself; said she scarcely felt her pains. But after inhaling it a few times she complained of nausea, and asked for something to vomit in; a vessel was handed her, and she vomited moderately. She soon after called for the chloroform again, and took the sponge herself. She would hold it first to one nostril and then to the other, snuffing it as eagerly as an old snuff-taker would a first rate pinch of snuff. At times she complained that I did not give her enough of it, After ten minutes had passed in this way, she seemed a little wild and hysterical; talked of her husband as being confined somewhere, and of her trust in me to let him out; she even seemed disposed to weep about it. I discontinued the chloroform for one or two pains, (which continued just as frequently and forcibly as before she had breathed of it at all,) when she really complained so much of my not giving it to her, that I again allowed it. It was during this time that I directed a cup of tea for her. I, myself, poured it from the cup into the saucer, while she raised herself on to her elbow and drank of it until the cup was emptied.

All this time I certainly supposed her to be partially sensible of her pains; but only partially so, for I could see plainly enough by her actions, that after a few inhalations, just as the pain was coming on, she did not suffer that sharpness of pain which she did when not under its influence; and she was herself continually saying how it relieved her pain, that she did not suffer from them as before.

The labor went on very rapidly. It really seemed that the dilatation was more rapid than it would have been withont the chloroform, although of this there must be doubt. At 10 1-2 o'clock there being full dilutation I ruptured the membranes. In five minutes more the head had got low down into the cavity of the pelvis, when I asked her to turn from her back on to her side. This she did immediately without any assistance.

She still continued in distress about her husband, fancying that he was about to desert her; said something about our destroying the child, and continued to make considerable outcry during the pains, although the chloroform was given her every pain. I had no doubt in my mind of her sensibility to all that was going on. Finally at 1-4 to 11, the child was born. It was her second child, was quite small, and according to her reckoning was one month premature.

There were but very moderate after-pains; no flooding. She did not sleep any that night; had a constant headache for which she kept her head bathed in spirits of camphor. The want of sleep nights was usual to her for some weeks before; the headache was not common.

The diarhoea returned the next day, but was finally subdued in three or four days, and she soon after got well and hearty.

On careful enquiry the next day, I found that she recollected scarcely anything from the first moment of breathing the chloroform; neither of the nausea and vomiting, nor of taking the cup of tea, nor of her turning on her side, at my request, shortly before the birth of the child, nor of the delivery of the child itself. As already remarked, I did not at the time think her wholly insensible; she heard readily; answered, generally, correctly; and did at once what was desired of her; and besides, called repeatedly for more chloroform. She recollected every thing done and said from two or three minutes after the child was born. Of all cases I have seen, this appears to me to be the most curious and interesting. I feel no doubt of her having told me the truth, when she denied all recollection of the labor after commencing the use of chloroform.

7. Mrs. J.—In labor with her second child; presentation 1st position of the vertex. The pains accompanying the dilatation of the os uteri were very feeble and at long intervals, so that the dilatation occupied altogether some twenty-four hours. The membranes ruptured immediately on the completion of the dilatation, and three forcible pains completed the delivery. It was only during the last two pains that chloroform was administered. It produced the usual effect of insensibility to pain. She knew when the child was being born, but her mind seemed to be employed upon something else, for while the child was passing into the world, she exclaimed,

"oh, it is pretty, it is pretty." I asked her afterwards what she was dreaming of. She said she saw something, she did not know what, that was very beautiful, "it was yellow and green." The getting up was rapid.

8. Mrs. Y.—In labor with her second child. The former labor had been tedious and painful, especially the second stage. On visiting her, I found the os uteri dilated, but the head rather indisposed to enter the superior strait. The waters soon afterwards escaped, yet there was but slight advance of the head. The pains were rapid, there being only from three to five minutes interval between them, yet inefficient. The patient was impatient under them; throwing herself about the bed and indisposed to take hold of anything to hold on to, or to pull, during the pains, and would not "hold in her breath" and bear down. Three-fourths of an hour passed in this way, without advancing the labor. The head had fairly engaged in the superior strait, but lying more against the right ilia, than presenting in the centre of the strait. I put about half a teaspoonfull of chloroform on to the sponge and had her inhale it on the accession of a pain. The next pain she breathed of it still more freely, and instantaneously as it were, there was a change in her behavior. She took hold of the sheet I had had tied to the bed post, and pulled most powerfully (for she was a strong hearty woman,) whenever the pains came on. Slight hysterical symptoms were observable, such as a disposition to weep, and otherwise acting as if she felt distressed about something. She, three or four times, asked me how long I thought it would be before she would get through, and repeated the question only the last pain but one, before the child was born. Not over a minute after the child was born, and while it lay upon the bed crying, she suddenly started and asked quickly, "what is the matter, what is the matter?" raised her head and looking around added, "why I have been asleep, hav'nt I?" She supposed the crying which woke her, was by her little girl. She declared she did not know when the child was born; nor was she sensible of any pain after the second inhalation of the chloroform; nor has she any recollection of asking me when I thought she would get through. The time from the commencement of the administration of the chloroform until the birth of the child, was twenty minutes. During this time, whenever I noticed a pain coming on, or whenever she showed by her cries (for she cried out some nearly every pain) or actions any apparent increase of distress, I directed the nurse to hold the sponge to her nostrils for three or four respirations. The recovery was rapid and without any bad symptoms.

In this case, also, I did not suppose her so entirely insensible to pain as she afterwards declared herself to have been. The apparent suffering, although slight compared to what it had been; the free use she made of the voluntary muscles; and the question she so often asked me, certainly indicated, at least, partial sensibility to her situation. In some respects, it will be noticed that this resembles the sixth case.

This concludes the first division of the cases. There is a close resemblance in the effects of chloroform upon the system in the *first*, *second* and *fifth* cases. There were in these complete anæsthesia, unaccompanied with any mental aberration, and nearly entire abrogation of the contractile power of the voluntary muscles. The *third* closely resembles them, except in the slight delirium which accompanied it. The *fourth* case presents the anomaly of the tolerable free use of chloroform failing to produce any very sensible effect over the system for better or for worse. The second trial with it succeeded better, by lessening the natural expressions of pain, without stopping them, while it destroyed all recollection of any pain. The bystanders feel as though the patient were in pain, and duly sympathize with her, while she herself feels none.

The *sixth* case is rather anomalous. I can easily fancy that it would be far more difficult for those who were by, to believe that she was insensible to all that was said and done by herself, as well as by others, than it would be for those to whom the case is related. The *seventh* case shows the curious fact, noticed also by others, of pleasant sights, dreams, or emotions occupying the mind of the patient while under the influence of this medicine. In the *eighth* case, the labor was nnequivocally hastened, I believe, by the inhalation of the chloroform. In the condition of the mind it shows some resemblance to the sixth case.

I now proceed to the narration of the cases of the second division; those in which a perfect knowledge of everything going on was not lost, or only momentarily so. There are seven cases in this class. Their consideration will not take much time, as we have but one relation in which to observe or study them, viz: *the possibility of exhibiting chloroform to a point short of causing insensibility, or in any way interfering with the full use of all her faculties, or with the full play of the voluntary muscles, and yet so benumbing the pain as to make it quite easily bearable.*

If this point can be established by experience, it would be, I believe, a great desideratum in midwifery, as far as the use of anæsthetic agents is concerned. In the *first* place, there would be no division of opinion as to its entire safety, when proper to use it at all; *secondly*, it would obviate an objection constantly met with among the women; that is, their dislike to be put in a condition of unconsciousness during child-birth, even if it be perfectly safe; they want to know all that is going on at such times.

9. Mrs. P.—In labor with her second child. First position of the vertex. I administered the chloroform at her particular request. I commenced its inhalation before the full dilatation of the os uteri, on account of her suffering from the severity of the pains. It was given in small doses almost every pain for just three-fourths of an hour. She would tell us when she first felt the pain coming on, and call for the sponge; her husband who sat at the head of her bed with it, would immediately apply it to her nostrils, when five or six good inspirations, especially if the sponge had been recently wet, would bring her sufficiently under its influence to remove it, and let her work out the rest of the pain in some comfort. Twice she thinks she momentarily lost herself when breathing it. She said she felt very sensibly its benumbing effect over the pains; it seemed to take off their sharp edge, and make them quite bearable. Not a single unfavorable symptom followed its use.

10. Mrs. R.—This patient consented to breathe it only at the eleventh hour; that is, not until the child was about being born. She got enough under its influence not to complain of the last pain or two, but lay quite comfortable, using the voluntary muscles to their full extent.

11. Mrs. L.—In labor with her first child. When I arrived the head had nearly reached the inferior strait; the os uteri had a diameter of about three inches; the membranes entire; the pains were at long intervals for such an advance of the head, from eight to ten minutes intervening between them; they were, however, tolerably sharp. The patient wished to breathe the chloroform if it would ease her pain. The first time she inhaled with great caution; with the second pain, quite freely; when the next pain came on, I did not offer it to her until she called for it, upbraiding me for negligence. She insisted, afterwards, upon having it on the accession of each pain, and it was with some difficulty that I could persuade her, two or three times, to have a pain or two without breathing it. It was one and a half hours from the time she first commenced inhaling it, before the birth of the child. The pains continued at long intervals until the very last. She did not lose her consciousness for one moment, yet in every instance, expressed relief from the inhalations.

12. Mrs. W.—Second child. She inhaled it only for about fifteen minutes during the latter part of the second stage. She was sufficiently under its influence that the birth of the child caused very little pain, yet was entirely conscious. I should think she was more under its influence at that moment, than were either of the three preceding cases at the corresponding time in their labors.

13. Mrs. B.—In labor with her first child. Her friends were unwilling that she should take chloroform, although willing herself. Its use was thereby postponed until the last moments of the labor. The head had reached the inferior strait, where it was delayed from the want of pain sufficiently expulsive; she could not bear down on account of a severe cutting pain such exertion gave her, in and through the central part of the abdomen, (in the fundus uteri, I supposed,) which was very distressing, and effectually prevented her from exerting herself. The head had lain in this position without gaining any, full three-fourths of an hour, the pains occurring once in five or six minutes, sharp and annoying. Her friends finally consented that she might try chloroform. The first inhalation "took away," as she expressed it, that pain in the bowels which had been so distressing; she bore down well, and after four or five pains the child was safely delivered. This is a case like the eighth, where the labor was decidedly advanced by the use of chloroform.

14. Mrs. P.—Third child. The labor was in every way natural. Chloroform was given during the last half hour, merely to relieve pain; and given in small and frequently repeated doses, so as not to cause a loss of sensibility. It was entirely effectual.

15. Mrs. J.—The first labor. I was called at 9 A. M., and found she had been in pain all night. Os uteri only half an inch in diameter. The pains recurring at short intervals until 11 o'clock, with very little progress. I bled her—took 18oz. The dilatation still proceeded slowly. The pains had become more sharp by six o'clock; os uteri dilated to two inches in diameter. I soon after commenced giving the chloroform, while she was sitting up, with the idea principally of relieving pain, and in the hopes of assisting in the dilatation of the os uteri, if the remedy had any such power, a point which my observations do not enable me to determine satisfactorily to my own mind. After inhaling it on the accession of each pain for nearly half an hour, she complained of being faint and asked for water; I attributed the faintness to the combined effects of fatigue, the bleeding, and the chloroform. The breathing of the latter was discontinued; not long afterwards she lay down again. The dilatation, I thought, had been more rapid than before, for the waters soon discharged, and the head began to press upon the perineum. The pains became more forcing, and I again gave the chloroform in tolerably free doses for two or three pains, when the child was born, as in the other cases, without suffering while under the influence of the remedy, and without loss of consciousness.

This concludes my list of cases. On looking them over for untoward symptoms which the chloroform *may* have had an agency in producing, I find in the *second* case, nausea, some dizziness, and a sense of thoracic oppression; in the *sixth*, one turn of vomiting while under its influence, and headache after coming out from it; and in the *fifteenth* case, the faintness was probably, in part, due to the same cause.

In the *third, sixth, seventh* and *eighth* cases, there were slight hysterical symptoms, talking, confusion of mind and ideas; in the *third, sixth* and *eighth*, the hallucinations were unpleasant, while in the *sixth* they were apparently very agreeable.

The *eighth* and *thirteenth* cases were the only ones in which I was fully satisfied that the chloroform had a decided influence in shortening the duration of the labor.

In the *fourth, sixth* and *eighth* cases, there were such positive expressions of pain, with apparent partial consciousness, that only the repeated declarations of the women could well convince me that they actually felt no pain.

And here let me remark upon the great caution that ought to be exercised in the administration of chloroform in similar cases. Their actions and cries are so indicative of suffering, that we, the physician and assistants, have no doubt that they are partially sensible; and, perhaps; desirous of producing a decided effect, we cause them to inhale the chloroform in freer doses, and for a longer time; and what is the result? possibly, death. If the patient is of such a constitution that a moderate dose produces insensibility, we are satisfied, and do not urge it; but when with equal insensibility, there is an apparent consciousness of suffering, complicated with a semi-delirious condition of the mind, here it is that we might err, and without a proper knowledge of this important fact, and caution in the use of our remedy, we might do irreparable injury. Never, while in the practice of medicine, have I felt my responsibility greater than while reflecting upon these cases; and feeling how easy it might have been, to have done harm, if, urged by a desire for a decided effect and a brilliant result, (as the safe delivery of a patient without the appearance or expression of pain really appears to be) I had urged the chloroform until complete anæsthesia was produced.

I commenced the use of chloroform, decidedly influenced by the opinion of Prof. Simpson, that it was better and safer to give a full dose, and at once produce a decided effect, than to give it in smaller quantities, repeated as occasion might require; and although my cautiousness did not always allow me to reach that point at once, I persevered, in the first few cases, in giving larger and larger doses at every pain, until I had produced that result.

With my present opinions and experience, I should, and have, of late, done differently. So far from desiring that a patient should lie in a state, either of entire unconsciousness, or of dreamy insensibility in the intervals of pain, I infinitely prefer that she should be entirely conscious; some how I feel much safer and less nervous about her; I then have no fears of a possible bad result.

I think that the results afforded by the second set of cases, fully demonstrate the proposition with which I commenced their narration, viz: the possibility of exhibiting chloroform to a point short of causing unconsciousness, or in anywise interfering with the full use of all her faculties, or the full play of the voluntary muscles; and yet so benumbing the pain as to make it quite easily to be borne. I would add that we can anticipate this result, not only in occasional cases, but I believe, also, in the large majority of cases.

I would be clearly understood, that in all these cases of the second division, there were expressions of pain or uneasiness with each contraction of the womb; some more and some less; the voluntary muscles were, in all, excited into full action, as is usual when chloroform has not been exhibited. I depend upon two facts to determine the relief they received; 1st, the contrast apparent to all between their suffering before breathing chloroform, and that while under its influence; 2d, their own declarations of decided relief from its use; and their positive assertions that if it be their destiny to have more children, they would be sure to have it again.

The *fifteenth* case was the least satisfactory, in this respect, of any of the second class; being the only one which did not call for it after having once breathed it, if we happened to be a little dilatory in giving it to them on the recurrence of a pain, or allowed the sponge to get emptied of it.

As to my manner of giving chloroform in obstetrical cases; I do not now speak of surgical or medical cases. I prefer administering it in small or moderate doses. My sponge will hold just one fluid drachm of the chloroform, when filled to saturation; this I call a full dose, and I can be in no danger of incautiously giving more. From thirty to forty drops, I call a moderate dose; from fifteen to twenty-five, a small one. From five or six to ten or twelve *free, deep* inspirations from the sponge, wet with a moderate dose, will generally bring a patient sufficiently under its influence to benumb pain, and even to cause momentary insensibility; and if the sponge should be wet at every pain, and the inspirations be likewise free, full anæsthesia cannot fail to be soon brought on. I generally make one wetting of the sponge do for two or three pains. I prevent evaporation by having it loosely stitched to the centre of a piece of oiled silk, of about six inches square, which silk is loosely wrapped around the sponge when taken from the face; and covers lightly the nose, mouth and cheeks of the patient while she is inhaling the chloroform. It ought not at such times to be tightly held over the nose but loosely so as to allow air to enter from under its edges, sufficiently moderately, to dilute the chloroform.

I have a perfect repugnance to administering it by taking a pocket-handkerchief, or a large sponge, and, at random, pouring from one to three or four teaspoonfuls on it for the patient to breathe. I once saw a man indirectly destroyed by putting out morphine, for him, in powders without weighing it. What wonder in having a similar result from chloroform, from similar carelessness. Both are remedies of activity and power.

I have some ideas upon the value the different symptoms seen in the patient while under the influence of chloroform, ought to have upon our mind in judging of her true condition, and, of course, upon our use of the remedy; but as they are as yet imperfect from want of sufficient observation, I forbear to trouble the reader with them.

Buffalo, Oct., 1848.

